# Flaking process increases the NF-κB inhibition activity and melanoidin extractability of coffee

**DOI:** 10.1002/fsn3.19

**Published:** 2012-12-26

**Authors:** Yi-Fang Chu, Kang Hu, Thomas Hatzold, Richard M Black, Don Chen

**Affiliations:** 1 Kraft Foods Global BrandsLLC 801 Waukegan Road, Glenview, Illinois, 60025; 2 Feinberg School of Medicine, Northwestern UniversityChicago, Illinois, 60611

**Keywords:** Coffee, flaking, melanoidin, nuclear factor-kappa B inhibition

## Abstract

Research on the health impacts of coffee has escalated. However, few studies were devoted to understanding the potential impact of mechanical processing on coffee's chemistry and subsequent health implications. Coffee flaking is a commonly used process to improve extractability and aroma characteristics. In this study, we studied the biochemical activity, chemical composition, and microstructure of coffee before and after flaking. We found that flaked coffee extract had 3.3-fold higher activity in inhibiting nuclear factor-kappa B (NF-κB) activation than regular coffee extract. Interestingly, flaking did not significantly alter the amount of coffee phenolics. It increased coffee melanoidin, by 2.1-fold, which likely contributed to the observed higher activity in inhibiting NF-κB activation. Flaking crushed cell walls revealed by microscopy might possibly result in disruption of polysaccharide entanglement and release of high-molecular-weight compounds, such as melanoidins. Consequently, the increased melanoidin content in the brew resulted in the increased inhibition of NF-κB activation. Small molecules, like coffee phenolics, are readily soluble in water during coffee brewing even without flaking, suggesting that flaking has no effect on its extractability. In summary, our investigation revealed that flaking enhanced NF-κB inhibition activity, possibly through the release of melanoidins from crushed cell microstructures.

## Introduction

Coffee consumption represents a significant proportion of daily beverage intake in Western countries (Pulido et al. 2003; Svilaas et al. 2004; Tunnicliffe and Shearer 2008; Nkondjock 2009). More and more research has indicated correlations between coffee consumption and health benefits. The anti-inflammatory and antioxidant activities of coffee have been hypothesized to be its major mechanism of actions (De Paulis et al. 2002; Pereira et al. 2006; Chu et al. 2011). Consequently, much research has been directed in identifying chemical compounds in coffee and characterizing their biological activities (Farah et al. 2006; Pereira et al. 2006; Chu et al. 2009, 2011).

Flaking is a widely used technology to process roasted coffee beans into thin pieces. For decades, flaking has been used by manufacturers to increase coffee's extractability and flavor profile. To optimize efficiency while maintaining a coffee product with superior extractability and low acidity, flake thickness, particle-size distribution, bulk density and moisture content have been studied and optimized. Increased coffee extractability makes flaking highly desirable in the food industry. However, the effects of flaking on the biological activities of the resulting coffee brew remain unknown.

In previous works, we studied coffee phenolics and melanoidins, two major coffee chemical groups (Borrelli et al. 2002; Moreira et al. 2005), and their respective biological activities, including antioxidant effects and inhibition of nuclear factor-kappa B (NF-κB) activation in a cell model (Chu et al. 2009, 2011; Chen et al. 2011). Chlorogenic acids, a group of hydroxycinnamic acids, are well known as coffee bioactives. Coffee chlorogenic acid consists primarily of caffeoylquinic acids, feruloylquinic acids, dicaffeoylquinic acids, and, in smaller amounts, *p*-coumaroylquinic acids and their derivates (Michael 1999). The melanoidins are a group of water-soluble, high-molecular-weight brown polymers formed via the Maillard reaction, a type of non-enzymatic browning (Fogliano and Morales 2011). This reaction occurs when high temperatures and specific water activity are present during coffee roasting, where carbonyl groups of reducing sugars condense with amino groups of peptides (Friedman 1996; Borrelli et al. 2002; Bekedam et al. 2006). Melanoidins in roasted coffee are known to contribute to the color and distinct aroma of coffee (Hofmann et al. 2001).

In this study, we investigated the effects of flaking on the biological activity of the resulting coffee brew using the previously established inhibition of NF-κB activation model (Chen et al. 2011; Chu et al. 2011). We also analyzed the chemical profiles of coffee before and after flaking and explored the correlation between changes in chemistry and biological activity.

## Materials and Methods

### Materials

Reagents were purchased from Sigma (St. Louis, MO) unless otherwise specified. High-performance liquid chromatography (HPLC)-grade water (EMD Chemicals, Gibbstown, NJ), acetonitrile, and methanol were obtained from Fisher Scientific (Boston, MA). American Chemical Society-grade formic acid was obtained from EMD Chemicals. A luciferase assay kit was purchased from Promega (Madison, WI). Myoblast C2C12 cells with a stably transfected NF-κB luciferase reporter gene were obtained from Panomics (Fremont, CA). AAPH (2,2′-azobis [2-amidinopropane] dihydrochloride) was purchased from Wako Chemicals USA (Richmond, VA). Fluorescein (sodium salt) and Trolox (6-hydroxy-2,5,7,8-tetramethylchroman-2-carboxylic acid) were obtained from Aldrich (Milwaukee, WI). Randomly methylated β-cyclodextrin (Trappsol, Pharm Grade, RMCD) was obtained from Cyclodextrin Technologies Development Inc. (High Springs, FL). Formic acid (American Chemical Society reagent grade) was obtained from EMD.

### Coffee preparation

Commercially available Yuban 11 oz roasted and ground coffee was used as the raw material and the control (approximately 50% of the particles are of the size of 1000 μm). Flaking was conducted using a 18 × 12 Hydraulic Dual Drive Roller Mill (Ferrell-Ross, Hereford, TX) at room temperature, with a roll speed in the range of 150–300 rpm, and a roll gap between 2 and 20 mil (1 mil = 1/1000 inch). After flaking, both control and flaked coffees were brewed using a Mr. Coffee 12-cup machine with a coffee-to-water ratio of 1:48.4 (g/mL). Coffee brews were freeze-dried using a mini freeze drier (Thermo Fisher Powerdry L3000, Waltham, MA). These freeze-dried coffee samples were used for chemical, biochemical, and cell culture characterization.

### Measurement of NF-κB activation and cell viability

The effects of coffee on NF-κB activation were measured with myoblast C2C12 cells stably transfected with a NF-κB luciferase reporter gene (Panomics). Cells were grown in Dulbecco's Modified Eagle Medium containing 10% fetal bovine serum, 2 mmol/L l-glutamine, and 100 μg/mL hygromycin at 37°C in a humidified 5% CO_2_ incubator. Approximately 5 × 10^4^ cells were seeded in 96-well plates overnight before the medium was replaced with serum-free medium. Cells (triplicate wells) were incubated with coffee samples (0–0.25 mg/mL, in serum-free medium) or stimulated with TNF-α (20 ng/well) for 4 h, and luciferase activity was measured using a luciferase assay kit (Promega; Fang et al. 2008).

Cell viability was tested by quantification of cellular ATP levels using the luciferase/luciferin assay. Cells were cultured in serum-free medium for 4 h in triplicates. TNF-α (50 ng/well) and serially diluted coffee samples were added to each well and incubated for 1 h, and luciferase activity was measured using a luciferase assay kit (Promega; Fang et al. 2008).

Luciferase activity readings were made using a Synergy 2 plate reader (BioTek, Winooski, VT); IC_50_ values were calculated using Excel (Microsoft, Redmond, WA).

### Particle morphology

The morphology of control and flaked particles was assessed using a FEI Quanta 400F scanning electron microscope (FEI, Hillsboro, OR). Samples were randomly selected and positioned on stage without further treatment. The scanning was conducted at Low-Vacuum mode with vacuum 0.75 Torr in chamber. Resolution was 1.5 nm at 30 kV (SE). Images were processed using the system software and displayed as 4-quadrant.

### Oxygen radical absorbance capacity assays

To measure oxygen radical absorbance capacity (ORAC) values in the hydrophilic fraction (ORAC_hydro_), coffee samples (5 g) were extracted with acetone/water (20 mL, 50:50 v/v) using an orbital shaker at room temperature for 1 h. The mixtures were centrifuged (1972*g*, 10 min, 4°C; Rotanta 460R centrifuge; GMI, Ramsey, MN). The ORAC_hydro_ values of the supernatants were measured using a method adapted from Ou et al. (2001), using a FL600 plate fluorescence reader (Bio-Tek Instruments, Inc., Winooski, VT) using the sKC4 3.0 software. The excitation wavelength was set at 485 nm and the emission wavelength at 530 nm. To measure ORAC values in the lipophilic fraction (ORAC_lipo_), freeze-dried brewed coffee samples (5 g) were extracted with hexane/dichloromethane (10 mL, 50:50 v/v) twice. ORAC values of the combined organic phase were measured using a previously published method (Huang et al. 2002; Wu et al. 2004). Triplicate samples were measured in both ORAC assays.

### Total phenolics

Total phenolic content was determined according to the method of Singleton and Rossi (1965). Briefly, 1.0 mL gallic acid standard or the previously lyophilized coffee extract was mixed with 15.0 mL water and 1.0 mL Folin–Ciocalteu reagent, and incubated at room temperature for 10 min. After adding 20% sodium carbonate (3.0 mL) and incubating at 40°C for 20 min, absorbance was measured at 755 nm using an Agilent 8453 ultraviolet (UV)-visible spectrophotometer (Waldbronn, Germany). Samples were measured in triplicate. Total phenolic content was expressed as milligrams of gallic acid equivalent per gram of the lyophilized sample.

### Quantitative analysis of chlorogenic acids and lactones

Coffee samples (0.1 g) were extracted with 12 mL methanol. Mixtures were sonicated at room temperature for 10 min, followed by shaking at 300 rpm for 30 min. After centrifugation (1972*g*, 10 min, 4°C; Rotanta 460R centrifuge), supernatants were analyzed using a Shimadzu HPLC system (Nakagyo-ku, Kyoto, Japan) (pump: LC-20AT, auto sampler: SIL-HTC, UV detector: SPD-20A) equipped with a 4.6 × 250-mm Symmetry Shield-C18 (5 μm) column (Waters, Milford, MA). The mobile phase consisted of H_2_O containing 0.4% formic acid (A) and acetonitrile (B). Separation was carried out at room temperature at a flow rate of 0.8 mL/min. The percentage of B in the mobile phase was increased from 0% to 50% over 40 min, then increased to 95% over 5 min, and maintained at 95% for a further 5 min. The percentage of B in the mobile phase was then decreased to 0% in 3 min, and maintained at 0% for 5 min for column reequilibration. A SCIEX 4000 Q-trap detector (Framingham, MA) with electrospray ionization source in positive mode was used to monitor ions at *m/z* 355, 369, 321, 337, 351, 499, and 517. Identification of caffeoylquinic acids, dicaffeoylquinic acids, feruloylquinic acids, and their lactone derivatives was achieved by comparison of mass spectrometry data with literature values (Farah et al. 2005; Perrone et al. 2008). Quantification of specific compounds separated by HPLC was based on UV detection at 325 nm using 5-caffeoylquinic acid as the reference (Kirsten Schrader and Ulrich 1996). Samples were measured in triplicate, and the acquired data were analyzed using the Analyst software (ver. 1.4.1, AB SCIEX, Framingham, MA).

### Gel filtration chromatography

Gel filtration chromatography was carried out at 40°C using a Shimadzu HPLC system (pump: LC-20AT, auto sampler: SIL-HTC, UV detector: SPD-20A) two 300 × 7.8-mm i.d. TSKgel columns in tandem (G4000 PWXL and G2500 PWXL; TosoHaas, Stuttgart, Germany), in combination with a PWX-guard column. The mobile phase (0.2 mol/L sodium nitrate) was maintained at 0.8 mL/min, and elution was monitored at 405 nm. The sample was dissolved in the mobile phase and centrifuged prior to injection (100 μL).

### Statistical analysis

Statistical analysis was performed on Excel (Microsoft) using Student *t*-test or ANOVA. The results are shown as the means + SEM. *P* < 0.05 was considered statistically significant.

## Results and Discussion

### Inhibition of NF-κB activation in flaked and regular coffee

**Figure 1:**
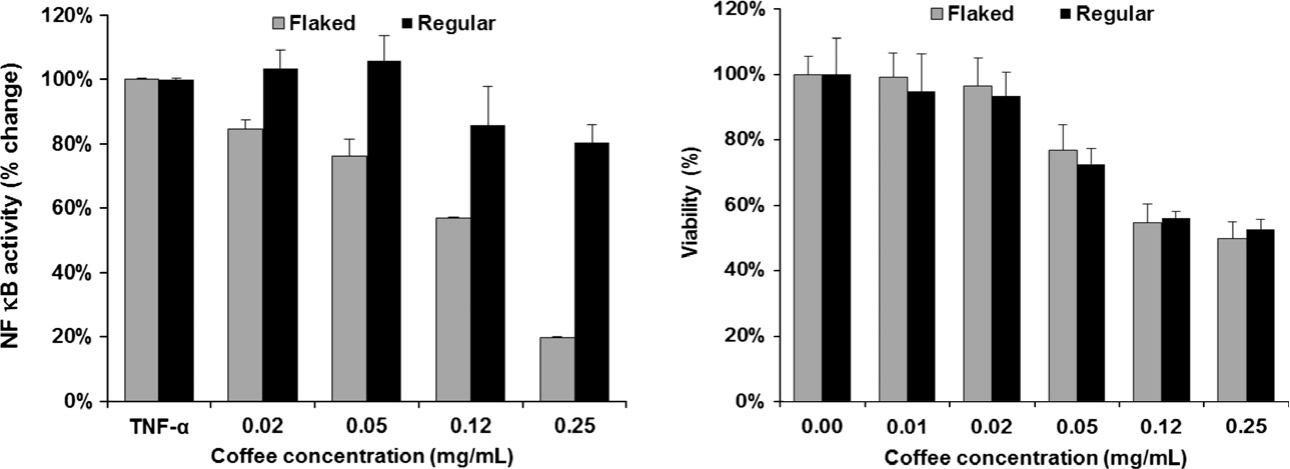
Nuclear factor-kappa B (NF-κB) activity and cell viability with regular and flaked coffee samples.

Nuclear factor-kappa B is an essential transcription factor in regulating cell signaling pathways, including cellular immune responses to stress (Yamamoto and Gaynor 2001) and inflammation (Roebuck 1999; Sethi et al. 2008). As shown in Figure 1, the flaked coffee decreased NF-κB activity across all sample concentrations, with maximum inhibition at 0.25 mg/mL, with a mild effect on cell viability. At this concentration, the flaked sample inhibited NF-κB activity by 80%, while the regular sample only inhibited it by 20%. The IC_50_ for regular coffee was 0.5 mg/mL and for flaked coffee was 0.15 mg/mL. These results indicate that the NF-κB inhibition of flaked coffee was 3.3-fold more potent than that of regular coffee. Quantification of cellular adenosine triphosphate levels using the luciferase–luciferin assay revealed that, under the conditions described, incubation of cells for 24 h with up to 0.05 mg/mL coffee did not decrease viability below 80%, showing that the coffee concentrations used in these experiments (0–0.05 mg/mL) were not toxic to the cells. As the concentration of coffee samples was increased from 0.05 to 0.25 mg/mL, cell viability was about 50%. Fortunately, similar cell viability was seen with both flaked and regular coffee samples (Fig. 1), which ensured that the inhibition of NF-κB activation by the flaked and regular coffee samples was measured under similar conditions and the data were comparable.

### Antioxidants and chlorogenic acids levels before and after flaking

Our previous studies indicate that both coffee phenolics and melanoidins inhibited NF-κB activation (Chen et al. 2011; Chu et al. 2011). To determine whether the observed difference in NF-κB inhibition between flaked and regular coffee was due to a difference in coffee phenolics and/or melanoidins, we analyzed phenolics in coffee before and after flaking. The results are listed in Table 1. It showed that antioxidants and small molecules, including phenolics and chlorogenic acids, in flaked coffee remained at levels relatively similar to those of regular coffee. Total phenolic and total chlorogenic acid levels were within a 20% range of the regular coffee values. Surprisingly, the flaking process did decrease the total chlorogenic acid level. As a consequence, there was also no major difference between the ORAC values of regular and flaked coffee (Table 1). The marginal difference in coffee phenolics in regular and flaked coffee suggests that coffee phenolics might not account for the observed difference in NF-κB inhibition between flaked and regular coffee as shown in Figure 1.

**Table 1:** Contents of coffee extracts from flaked and regular samples (antioxidants and small molecules, including phenolics and chlorogenic acids, were found at similar levels [±20%])

Contents	Flaked	Regular	F/R
ORAC_hydro_	18.44	22.53	0.82
ORAC_Lipo_	1.57	1.25	1.26
ORAC_Total_	20.01	23.78	0.84
Total phenolic	1.51	1.36	1.11
Total chlorogenic acids	705	902	0.78
3-CQA	132	107	1.23
4-CQA	216	332	0.65
5-CQA	161	170	0.95
3-CQL	12	16	0.75
3-FQA	ND	ND	ND
4-CQL	11	21	0.52
4-FQA	70	72	0.97
5-CQL	ND	ND	ND
5-FQA	36	35	1.03
3-FQL	ND	ND	ND
4-FQL	ND	ND	ND
5-FQL	ND	ND	ND
3,4diCQA	30	33	0.91
3,5diCQA	14	51	0.27
4,5diCQA	23	65	0.35

Data are the mean values of three independent measurements. SEMs were within ±15% of the means.

F/R, flaked/regular; ORAC, oxygen radical absorbance capacity; CQA, caffeoylquinic acid; FQL, feruloylquinic acid; ND, not detectable. ORAC: μmol/mL; phenolics: μmol/mL; chlorogenic acids and lactones: mg/L.

### Changes in melanoidin abundance and composition

**Figure 2:**
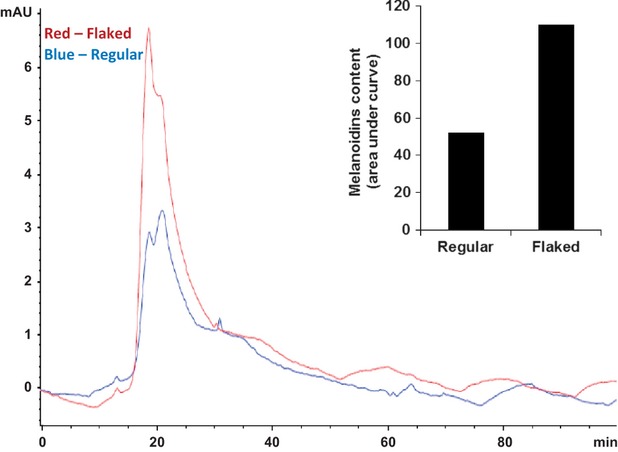
Melanoidin content in coffee. Melanoidin levels were increased 2.1-fold in flaked samples over regular samples.

Melanoidins comprise the majority of large molecules in coffee and can be separated from other components in coffee through gel filtration chromatography. With regard to molecular weights, coffee phenolics are usually small molecules, with molecular weights well below 1000 Da. In contrast, melanoidins have much higher molecular weights (Bekedam et al. 2006). Typically, melanoidin content can be measured by absorbance at 405 nm, with the specific extinction coefficient as a means of characterizing different coffee melanoidins (Borrelli et al. 2002; Bekedam et al. 2006). The chromatogram obtained by monitoring elution at 405 nm is shown in Figure 2. The red and blue peaks represent melanoidin readings in flaked and regular coffee, respectively. The signals are proportional to the concentration of melanoidin; the melanoidin content in flaked coffee (110) was consistently higher than that in regular coffee (52). Overall area under curve calculation showed that flaking increased melanoidin abundance by 2.1-fold. Our previous results showed that melanoidin has anti-inflammatory activity (Y. M. Chen and Y. F. Chu, unpubl. data). Thus, it is plausible that the difference in NF-κB inhibition may be explained by the difference in melanoidin contents between flaked and regular coffee (Fig. 2). However, given the complexity of both NF-κB activation and melanoidin composition, further studies were proposed to elucidate the mechanisms of action in more detail.

### Microstructure of coffee flaking

**Figure 3:**
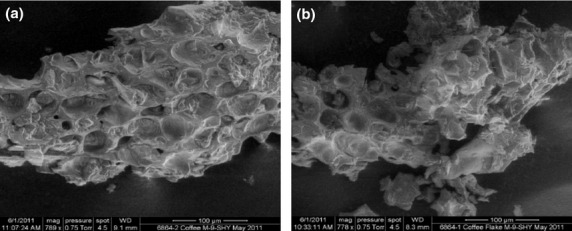
Structures of coffee before (a) and after (b) flaking. (a) Regular, M-9-SHY R&G, 760 μm. Most of the cells remained intact after regular grinding. (b) Flaked, M-9-SHY R&G at 4-mil (100 μm) gap and 250-rpm roll speed. A significant number of cells were crushed.

To further understand the effect of flaking on coffee microstructure and increase in the extractability of melanoidins, electron micrograph was conducted. The majority of coffee cell wall material is polysaccharides. The entanglement structure and low solubility make it difficult for the polymers to be extracted by brewing. From Figure 3, we observed that for flaked coffee, a significant amount of coffee cell wall has been crushed compared with the regular coffee. This suggests that the polysaccharide entanglement is disrupted, which favors the release of polymers or high-molecular-weight compounds, such as melanoidins. Consequently, the increased melanoidin content in the brew resulted in increased inhibition of NF-κB activation. Small molecules, like coffee phenolics, are readily soluble in water during coffee brewing even without flaking; suggesting flaking has no effect on their extractability.

## Conclusions

Recent developments to improve the efficiency and extractability in coffee brewing have prompted the use of flaking technique in manufacturing roast-and-ground coffee products. Our study provides an initial insight into the effect of flaking in increasing melanoidin content in the extract, thus leading to elevated melanoidin-associated inhibition of NF-κB activation.
